# ICTV Virus Taxonomy Profile: *Deltavirus*

**DOI:** 10.1099/jgv.0.001150

**Published:** 2018-10-12

**Authors:** Lars Magnius, John Taylor, William S. Mason, Camille Sureau, Paul Dény, Helene Norder, ICTV Report Consortium

**Affiliations:** ^1^​ Ulf Lundahls Foundation, 10061 Stockholm, Sweden; ^2^​ Fox Chase Cancer Center, Philadelphia, PA 19111, USA; ^3^​ Institut National de la Transfusion Sanguine (INTS), CNRS-INSERM U1134, Paris, France; ^4^​ Centre de Recherches en Cancérologie de Lyon, INSERM U1052, UMR CNRS 5286, Team Hepatocarcinogenesis and Viral Infection, Lyon, France; ^5^​ Department of Infectious Diseases, Institute of Biomedicine, Sahlgrenska Academy, University of Gothenburg, 41345 Gothenburg, Sweden

**Keywords:** *Deltavirus*, ICTV report, taxonomy, hepatitis delta virus, hepatitis, helper virus, hepatitis B virus

## Abstract

Hepatitis delta virus, the only member of the only species in the genus *Deltavirus*, is a unique human pathogen. Its ~1.7 kb circular negative-sense RNA genome encodes a protein, hepatitis delta antigen, which occurs in two forms, small and large, both with unique functions. Hepatitis delta virus uses host RNA polymerase II to replicate via double rolling circle RNA synthesis. Newly synthesized linear RNAs are circularized after autocatalytic cleavage and ligation. Hepatitis delta virus requires the envelope of the helper virus, hepatitis B virus (family *Hepadnaviridae*), to produce infectious particles. This is a summary of the International Committee on Taxonomy of Viruses (ICTV) Report on the taxonomy of *Deltavirus* which is available at www.ictv.global/report/deltavirus.

## Abbreviation

HDAg, hepatitis delta antigen.

## Virion

**Fig. 1.**
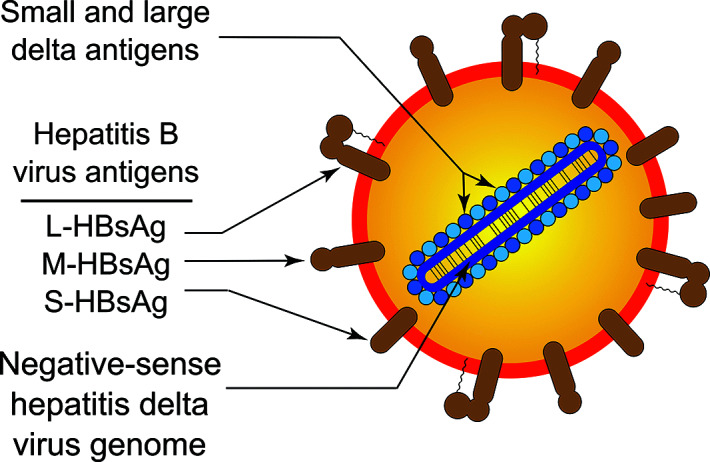
Schematic representation of a particle of hepatitis delta virus.

Virions of hepatitis delta virus are 36–43 nm spherical particles without visible surface projections (Table 1, Fig. 1). The lipid envelope contains three envelope proteins of the co-infecting human hepatitis B virus, and an inner ribonucleoprotein comprising the RNA genome of hepatitis delta virus and approximately 70 copies of the hepatitis delta virus-encoded protein, hepatitis delta antigen (HDAg) [1], which exists in small (S-HDAg, p24) and large (L-HDAg, p27) forms. L-HDAg differs from S-HDAg by a 19 amino acid carboxy-terminal extension. The ribonucleoprotein has variable quantities of S- and L-HDAg proteins associated with the genome RNA.

**Table 1.** Characteristics of the genus *Deltavirus*

Typical member:	hepatitis delta virus clade 1 (M21012), species *Hepatitis delta virus*, genus *Deltavirus*
Virion	36–43 nm in diameter with an outer envelope containing all three envelope proteins of the helper hepadnavirus and an inner ribonucleoprotein consisting of hepatitis delta virus negative-sense RNA and S- and L-HDAg proteins
Genome	Circular negative-sense single-stranded RNA of ~1.7 kb
Replication	Rolling circle mechanism of RNA-directed RNA synthesis by host RNA polymerase II and autocatalytic cleavage and recyclization in the nucleus
Translation	mRNA species for S- and L-HDAg proteins
Host range	Hepatitis B virus-infected humans are the only known natural hosts
Taxonomy	One genus including a single species

## Genome

**Fig. 2.**
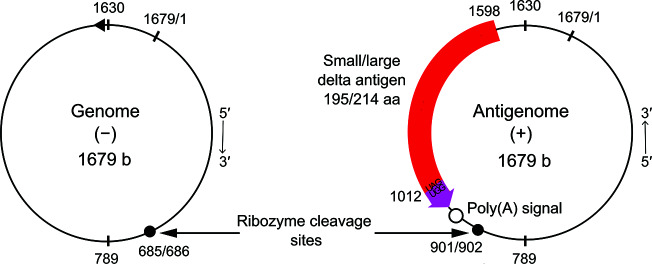
Organization of the genome and antigenome of hepatitis delta virus (M21012). Both RNAs are circular and have the ability to fold into an unbranched rod-like structure via intra-molecular base pairing, with the rod ends at positions 789 and 1630. Each RNA has a ribozyme, with the cleavage sites as indicated. Deamination of a UAG termination codon at the end of the small delta antigen ORF (red) results in the expression of a 19 aa extension (purple) to produce large delta antigen. The transcription start site on the genome strand at position 1630 is indicated by a triangle.

The genome consists of a single ~1.7 kb molecule of circular negative-sense single-stranded RNA (Fig. 2). Due to ∼70 % intramolecular base pairing it may fold on itself, forming an unbranched rod-like structure. Both genomic and antigenomic RNAs contain sequences with ribozyme functions that are capable of self-cleavage and possibly self-ligation [2], properties that make this genome unique and distinct from other animal viruses, although similar in these respects to viroids [3].

## Replication

Replication of hepatitis delta virus requires the presence of a hepadnavirus to provide envelope proteins; for this reason it can be regarded a satellite virus [3]. Natural infection with hepatitis delta virus only occurs in hepatitis B virus-infected humans. However, it can be transmitted to chimpanzees if accompanied by hepatitis B virus, and transmission to woodchucks has also been achieved by using woodchuck hepatitis virus as a helper virus. The mechanism of deltavirus entry into human hepatocytes appears to be similar to that used by hepadnaviruses. Both viruses require the S and the pre-S1 domains of the hepadnavirus envelope proteins for attachment to, and entry into, susceptible cells by pre-S1 binding to a cellular receptor, sodium taurocholate co-transporting polypeptide.

Genome replication involves RNA-directed RNA synthesis in the nucleus by host cell RNA polymerase II [4]. Transcription is thought to occur by a double rolling circle mechanism that generates greater than genome-length forms of antigenomic and genomic RNA, which undergo site-specific autocatalytic cleavage and ligation to generate circular genomic and antigenomic monomers. The antigenome contains the ORF for the 195 amino acid S-HDAg, which is translated from an ~800 to ~900 nt mRNA that is 5′-capped and 3′-polyadenylated. During replication, the mRNA for the L-HDAg is generated by an adenosine being converted into an inosine by the cellular enzyme ADAR1 (adenosine deaminase acting on RNA). This results in a stop codon being replaced by a tryptophan codon [5].

As hepatitis delta virus assembly requires the envelope proteins of a helper hepadnavirus, its assembly pathway is likely to overlap with that of the helper virus. L-HDAg must be present for delta antigen-containing particles to be released, while S-HDAg is packaged if present in the cell, but is not sufficient for particle formation.

## Taxonomy

The sole genus, *Deltavirus,* includes a single species, *Hepatitis delta virus,* with up to 40 % nucleotide variation between isolates of the member virus, hepatitis delta virus. There are eight clades with different geographical distributions.

## Resources

Full ICTV Online (10th) Report: www.ictv.global/report/deltavirus.
